# Circulating 3-carboxy-4-methyl-5-propyl-2-furanpropanoic acid (CMPF) levels are associated with hyperglycemia and β cell dysfunction in a Chinese population

**DOI:** 10.1038/s41598-017-03271-1

**Published:** 2017-06-08

**Authors:** Shan Zhang, Peihong Chen, Hua Jin, Jufen Yi, Xinmiao Xie, Meili Yang, Ting Gao, Lili Yang, Cheng Hu, Xueli Zhang, Xuemei Yu

**Affiliations:** 1 Department of Endocrinology and Metabolism, Diabetes Ward, Fengxian Central Hospital, Shanghai, China; 2 Department of Endocrinology and Metabolism, Third Clinical Medical College of Southern Medical University, Guangzhou, China; 3 Shanghai Diabetes Institute, Shanghai Key Laboratory of Diabetes Mellitus, Shanghai Clinical Center for Diabetes, Shanghai Jiao Tong University Affiliated Sixth People’s Hospital, Shanghai, China

## Abstract

Several recent clinical studies have suggested that the levels of circulating 3-carboxy-4-methyl-5-propyl-2-furanpropanoic acid (CMPF) are significantly higher in patients with gestational diabetes mellitus (GDM), impaired glucose tolerance (IGT), and type 2 diabetes mellitus (T2DM). This study recruited a total of 516 participants. The following patient populations were enrolled: 99 newly diagnosed cases with T2DM, 219 cases with prediabetes [82 with isolated impaired glucose tolerance (I − IGT), 66 with isolated impaired fasting glucose (I − IFG) and 71 with impaired glucose tolerance and impaired fasting glucose (IGT + IFG)], and 198 cases with normal glucose tolerance [NGT, including 99 first-degree relatives of type 2 diabetes patients (FDRs) and 99 non-FDRs]. We investigated the circulating CMPF levels in subjects with different glucose metabolism statuses and examined the potential link between CMPF and β cell function. Our results indicate that the serum CMPF levels were elevated in the prediabetes, T2DM, and FDRs groups compared to the NGT group. Additionally, the serum CMPF concentrations were independently and negatively associated with the triglyceride levels and Stumvoll first-phase insulin secretion index. Cumulatively, our findings suggest that the circulating CMPF levels can predict glycolipid metabolism disorders. Furthermore, elevated serum CMPF concentrations may determine hyperglycemia and β cell dysfunction.

## Introduction

The prevalence of diabetes continues to rise despite improvements in living standards and dramatic changes in lifestyle and environmental factors^1, 2^. Therefore, it is critical to discover methods of protecting β cell function and improving insulin sensitivity. Unfortunately, is not currently possible to reverse β cell function loss to prevent hyperglycemia and the chronic complications of diabetes^3^. As a result, it is important to explore the early biomarkers of diabetes and initiate interventions as soon as possible.

3-carboxy-4-methyl-5-propyl-2-furanpropanoic acid (CMPF) is an endogenous metabolite of furan fatty acids and a major uremic toxin^4^. Several recent clinical studies have reported that circulating CMPF levels were significantly higher in patients with gestational diabetes mellitus (GDM), impaired glucose tolerance (IGT), and type 2 diabetes mellitus (T2DM)^5–7^. Studies in rodent models have shown that elevated CMPF values impair glucose-stimulated insulin secretion, increase advanced glycation end products and oxidative stress, and impair insulin granule maturation to accelerate diabetes development^6^. CMPF treatment *in vitro* leads to mitochondrial dysfunction and reduces glucose-induced accumulation of ATP. Additionally, CMPF also alters the activity of relevant transcription factors and can impair β cell function^5^. Therefore, reducing the serum CMPF levels could protect β cells.

The incidence of T2DM is strongly linked to family history^8^. Previous studies of Danish twins indicate that genes play a predominant role in the etiology of abnormal glucose tolerance and suggest that the heritability of T2DM is 26%^9^. Insulin resistance, β cell dysfunction, or both conditions may precede the development of T2DM in first-degree relatives (FDRs)^10^. A prospective study demonstrated that FDRs have twice the risk of developing T2DM as the general population^11^. Furthermore, both age and family history are potentially involved in the pathophysiology of T2DM^12^.

The serum CMPF concentrations are unknown in FDRs, which is a group that is at high risk for developing T2DM. Moreover, there is limited evidence supporting the relationship between circulating CMPF levels and the secretion of human pancreatic β cells. In this study, we investigated the circulating CMPF levels of patients with different glucose metabolism statuses. We also evaluated the correlation between CMPF and glycolipid metabolism as well as the potential link between CMPF and β cell function.

## Results

### Characteristics of Study Participants

The anthropometric and laboratory parameters of the subgroups [normal glucose tolerance (NGT), prediabetes which includes isolated impaired glucose tolerance (I − IGT), impaired glucose tolerance and impaired fasting glucose (IGT + IFG) and isolated impaired fasting glucose (I − IFG) three groups, T2DM, and FDRs] are summarized in Table 1. The following parameters significantly differed (*p* < 0.05) among the four groups: age, systolic blood pressure, diastolic blood pressure, waist circumference, body mass index, free fatty acids, low-density lipoprotein cholesterol, triglycerides, total cholesterol, fasting and 2-h plasma glucose levels, HbA1c, fasting and 2-h insulin levels, HOMA-IR, HOMA-β, and the Stumvoll first- and second-phase insulin secretion index values. Participants in the prediabetes, T2DM, and FDRs groups had higher levels of FPG, fasting insulin, and HOMA-IR than the NGT group.

**Table 1:** Clinical characteristics of the NGT, T2DM, prediabetes, and FDR participants.

Variable	NGT (n = 99)	Prediabetes			T2DM (n = 99)	FDRs (n = 99)	*p-value*
		I − IGT (n = 82)	I − IFG (n = 66)	IFG + IGT (n = 71)			
Age (years)	57.328 (51.712, 62.189)	57.466 (49.370, 66.127)	57.268 (51.794, 65.745)	62.101 (55.383, 70.332)**	55.603 (51.810, 62.751)	50.000 (44.000, 54.000)**	<0.001
Body mass index (kg/m^2^)	25.298 ± 2.974	25.148 ± 3.736	25.073 ± 2.840	25.179 ± 3.944	25.296 ± 2.978	24.014 ± 2.534**	0.039
Systolic Blood Pressure (mmHg)	121.583 (116.250, 135.250)	130.000 (120.000, 142.000)	134.000 (123.000, 150,000)**	130.000 (124.000, 140.000)**	140.000 (120.333, 143.333)**	120.000 (110.000, 130.000)*	<0.001
Diastolic Blood Pressure (mmHg)	80.000 (75.000, 84.625)	80.000 (77.292, 88.500)	83.333 (80.000, 90.000)**	82.000 (80.000, 90.000)**	80.667 (80.000, 90.000)**	80.000 (70.000, 84.000)	<0.001
Waist Circumference (cm)	83.171 ± 8.294	84.320 ± 8.565	84.809 ± 7.297	85.576 ± 7.566	86.354 ± 9.044	81.378 ± 8.636	0.001
Total cholesterol (mM)	5.022 ± 0.882	4.959 ± 0.942	5.153 ± 1.076	5.474 ± 1.054	5.486 ± 0.969**	4.890 ± 0.964	<0.001
Triglycerides (mM)	1.600 (1.030, 2.100)	1.520 (0.950, 2.198)	1.365 (1.045, 1.860)	1.750 (1.210, 2.440)	1.750 (1.180, 2.740)*	1.050 (0.750, 1.580)**	<0.001
HDL-c (mM)	1.301 ± 0.329	1.236 ± 0.291	1.302 ± 0.245	1.260 ± 0.299	1.324 ± 0.323	1.237 ± 0.247	0.193
LDL-c (mM)	3.140 ± 0.642	3.078 ± 0.882	3.067 ± 0.832	3.399 ± 0.848	3.18 ± 0.793	2.799 ± 0.716**	<0.001
Free fatty acids (mM)	0.124 (0.081, 0.156)	0.515 (0.413, 0.639)**	0.452 (0.304, 0.603)**	0.472 (0.376, 0.650)	0.490 (0.373, 0.660)**	0.350 (0.235, 0.460)**	<0.001
FPG (mM)	5.020 (4.640, 5.390)	5.370 (5.200, 5.500)**	6.190 (5.868, 6.383)**	6.300 (6.100, 6.470)**	7.040 (6.100, 7.580)**	5.160 (4.900, 5.400)*	<0.001
2hPG (mM)	5.970 (4.900, 6.820)	8.755 (8.228, 9.415)**	6.370 (5.578, 6.983)	8.680 (8.240, 9.430)**	12.340 (11.275, 14.703)**	6.00 (5.400, 6.800)	<0.001
HbA1c (%)	5.600 (5.400, 5.900)	5.500 (5.275, 5.800)	5.600 (5.375, 5.800)	5.900 (5.600, 6.200)**	6.200 (5.800, 6.700)**	5.400 (5.200, 5.700)*	<0.001
FINS (mU/L)	5.660 (4.020, 7.960)	7.620 (5.100, 10.245)*	8.260 (5.455, 10.400)**	7.040 (5.393, 10.198)**	7.780 (4.830, 11.060)**	7.030 (5.610, 9.210)*	0.034
2hINS (mU/L)	26.330 (13.590, 47.250)	66.695 (47.088, 97.593)**	35.155 (21.668, 55.218)	56.350 (41.520, 97.715)**	51.350 (30.980, 94.020)**	35.595 (24.158, 55.818)*	<0.001
HOMA-β	77.561 (52.129, 122.743)	80.976 (55.170, 112.852)	57.323 (39.476, 80.030)**	50.478 (41.095, 73.958)**	47.784 (31.815, 83.400)**	88.429 (65.645, 114.021)	<0.001
HOMA-IR	1.260 (0.898, 1.891)	1.746 (1.158, 2.388)**	2.279 (1.575, 2.951)**	1.986 (1.493, 3.002)**	2.363 (1.566, 3.568)**	1.592 (1.262, 2.201)*	<0.001
Stumvoll 1st phase insulin secretion index	1021.647 (870.920, 1206.340)	832.922 (615.664, 1036.983)**	695.297 (562.822, 953.991)**	501.135 (363.275, 799.300)**	400.669 (169.862, 688.011)**	991.828 (868.158, 1182.782)	<0.001
Stumvoll 2nd phase insulin secretion index	268.441 (229.887, 314.435)	219.005 (173.289, 292.632)**	275.320 (225.513, 312.583)	200.240 (152.351, 296.648)**	181.988 (141.613, 292.209)**	274.018 (241.702, 324.365)	<0.001

Data are presented as the means ± SD or medians (interquartile range).

*Significantly different vs. NGT at *p* < 0.05; **significantly different vs. NGT at *p* < 0.01.

HDL-c: high-density lipoprotein cholesterol; LDL-c: low-density lipoprotein cholesterol; FPG: fasting plasma glucose; 2hPG: 2-h postprandial glucose; HbA1c: hemoglobin A1C; FINS: fasting insulin; 2hINS: 2-h postprandial insulin; HOMA-IR: homeostasis model assessment of insulin resistance; HOMA-β: homeostasis model assessment-β.

The normal reference values are the following:

Total cholesterol: <6.20 mM; Triglycerides: 0–1.7 mM; HDL-c: 1.03–2.05 mM; LDL-c: 2.6–4.9 mM; Free fatty acids: 0.1–0.45 mM; FPG: 2.9–5.6 mM; 2hPG: 2.9–7.7 mM; HbA1c: 4–6.1%; FINS: 3–25 mU/L; After oral administration of 75 g of anhydrous glucose, plasma insulin rose to a peak in 30–60 minutes, the peak value was 5–10 times greater than that of the baseline value, and after 3–4 hours, it returned to the basic level.

### Circulating CMPF levels

**Figure 1:**
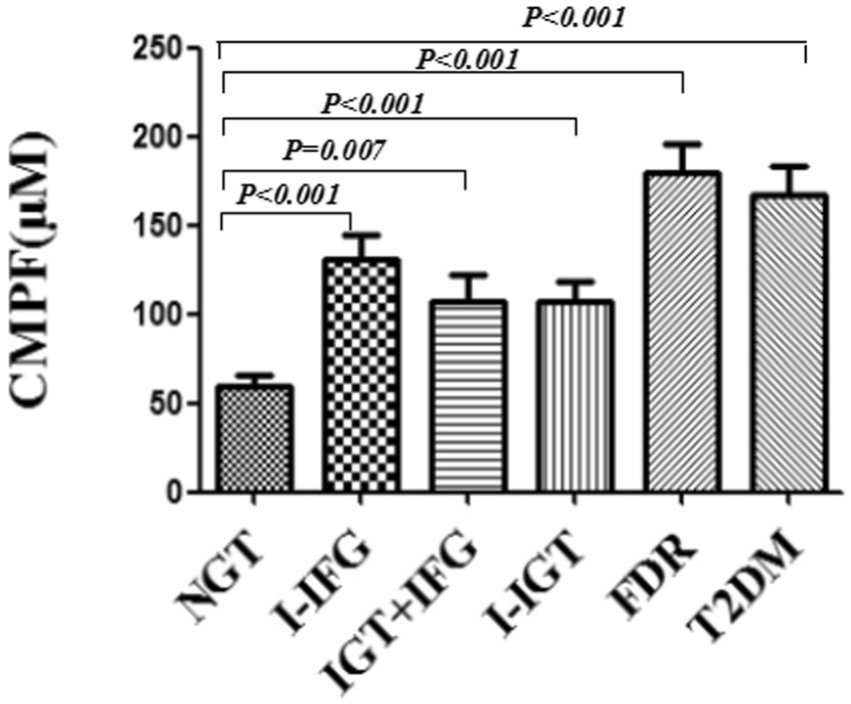
The CMPF levels in normal glucose tolerance (NGT), isolated impaired glucose tolerance (I − IGT), impaired glucose tolerance, and impaired fasting glucose (IGT + IFG), isolated impaired fasting glucose (I − IFG), type 2 diabetes mellitus (T2DM), and first-degree relatives of type 2 diabetes patients (FDR) participants. Compared with the NGT group serum levels, the CMPF levels were elevated in the I − IFG (*p* < 0.001), IGT + IFG (*p* = 0.007), I − IGT (*p* < 0.001), T2DM (*p* < 0.001) and FDRs (*p* < 0.001) groups.

The CMPF serum levels were elevated in the I − IGT (*p* < 0.001), I − IFG (*p* < 0.001), IGT + IFG (*p* = 0.007), T2DM (*p* < 0.001) and FDRs (*p* < 0.001) groups compared to the NGT group. The serum CMPF levels were higher in FDR participants than in the I − IGT participants (*p* < 0.001), I − IFG participants (*p* = 0.032) and IGT + IFG participants (*p* = 0.002). Therefore, our findings suggested that the FDRs group had higher CMPF levels than the prediabetes cases. However, within the prediabetes group, there were no differences observed in the CMPF levels compared to the I − IGT, I − IFG, or IGT + IFG groups (*p* = 0.131). There was no difference observed between the serum CMPF levels of the FDRs and T2DM groups (*p* = 0.352). Furthermore, there were no sex-based differences observed with respect to the serum CMPF levels (*p* = 0.564). The circulating CMPF levels of the six groups are presented in Fig. 1.

### Correlation between CMPF and clinical parameters

**Figure 2:**
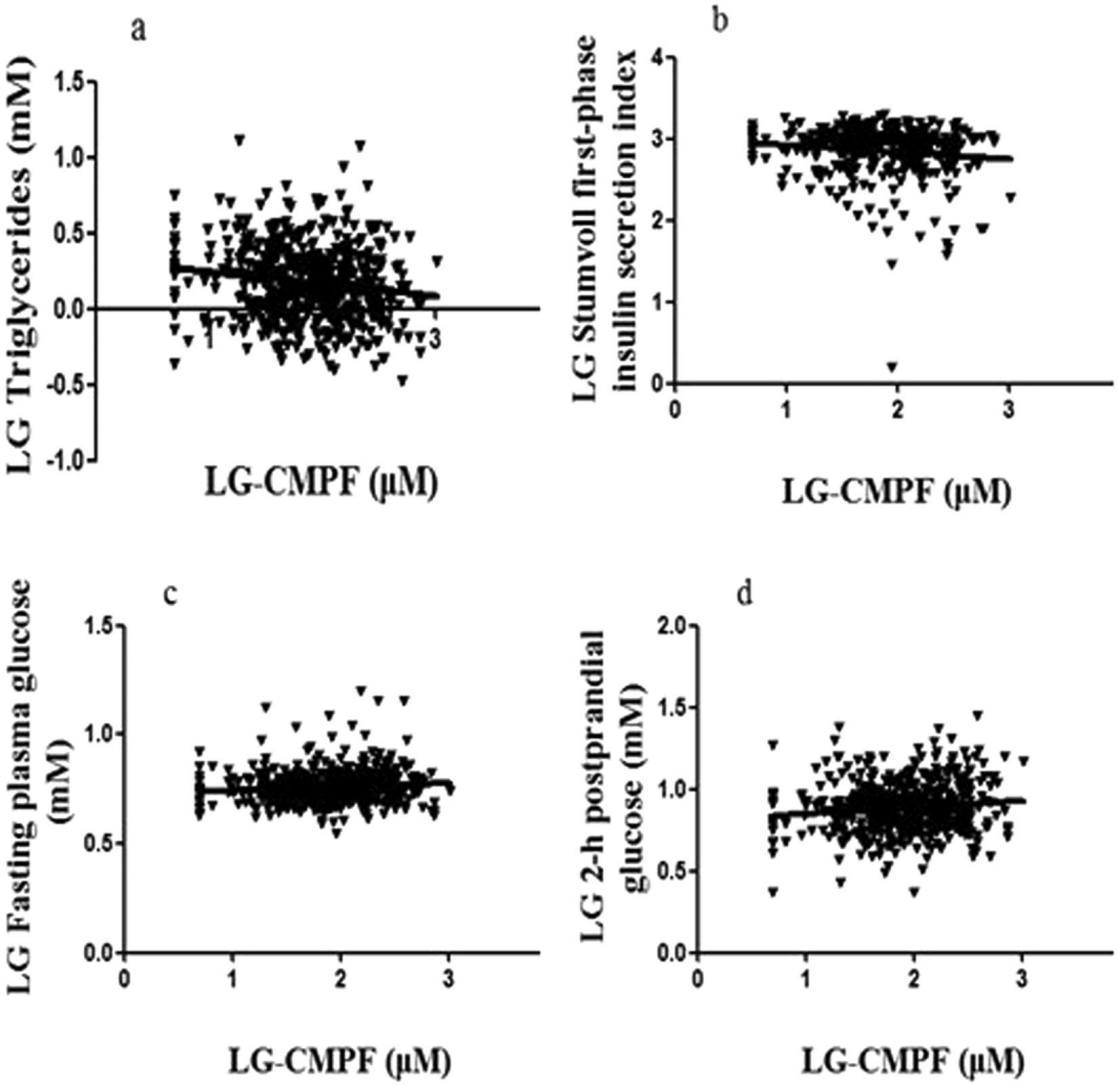
Correlation analyses between CMPF and body mass index, triglycerides, and the Stumvoll first-phase and second-phase insulin secretion index values. Pearson’s bivariate correlation analyses showed that the CMPF level was negatively correlated with triglycerides (*r* = −0.155, *p* < 0.001) and the Stumvoll first-phase insulin secretion index (*r* = −0.132, *p* = 0.010), but positively correlated with FPG (*r* = 0.104, *p* = 0.018) and 2hPG (*r* = 0.122, *p* = 0.005).

We conducted bivariate correlation analyses to analyze the relationship between circulating CMPF levels and various anthropometric parameters for all participants. The serum CMPF concentrations were negatively correlated with triglycerides (*r* = −0.155, *p* < 0.001) and the Stumvoll first-phase insulin secretion index (*r* = −0.132, *p* = 0.010). Conversely, the CMPF concentrations were positively correlated with FPG (*r* = 0.104, *p* = 0.018) and 2hPG (*r* = 0.122, *p* = 0.005). The correlation analyses between circulating CMPF and triglycerides, the Stumvoll first-phase insulin secretion index values, FPG, and 2hPG are presented in Fig. 2a–d respectively. A partial correlation analysis showed that the serum CMPF concentrations were positively correlated with FPG (*r* = 0.123, *p* = 0.005) and 2hPG (*r* = 0.145, *p* = 0.001) and negatively correlated with triglycerides (*r* = −0.137, *p* = 0.002) and the Stumvoll first-phase insulin secretion index (*r* = −0.144, *p* = 0.005) after adjusting for sex, age, and body mass index.

A multivariate stepwise linear regression analysis showed that the serum CMPF levels were independently associated with triglycerides (*β* = −0.395 ± 0.108, *p* < 0.001) and the Stumvoll first-phase insulin secretion index (*β* = −0.227 ± 0.080, *p* = 0.005) after adjusting for body mass index and the Stumvoll second-phase insulin secretion index (Table 2).

**Table 2:** A multiple stepwise linear regression analysis of the variables independently associated with serum CMPF levels.

	Β	SE	Standardized β	P
Triglycerides* (mM)	−0.395	0.108	−0.188	<0.001
Stumvoll first-phase insulin secretion index* (kg/m^2^)	−0.227	0.080	−0.145	0.005

*Log-transformed.

## Discussion

This study examined the serum CMPF concentrations in NGT, prediabetes, T2DM, and FDR participants. The results showed that the serum CMPF levels were significantly higher in the FDR group than in the NGT and prediabetes groups. However, there were no differences in the CMPF levels between the FDR and T2DM groups. The circulating CMPF levels were increased in participants with worse β cell secretion. Additionally, the circulating CMPF levels were negatively associated with triglycerides and positively associated with FPG and 2hPG. Cumulatively, these findings suggest that the CMPF levels can predict impaired glycolipid metabolism and that CMPF has an important role in diabetes development.

The relationship between circulating CMPF levels and diabetes has attracted significant attention. Prentice *et al*. first linked CMPF to glucose metabolism in a metabolomics study involving 24 women with GDM. The authors found that the serum CMPF levels were higher in GDM and prediabetes participants than in 24 controls^5^. Liu *et al*.^6^ reported patients with prediabetes or diabetes had greater serum CMPF concentrations than normal controls. A five-year follow-up assessment revealed that individuals with elevated CMPF levels at baseline were at higher risk of developing overt diabetes. A double-blind, parallel, and randomized controlled trial showed that 59 Chinese patients with T2DM had lower serum CMPF levels at baseline than healthy controls^13^. In the current study, we increased the patient sample size, and our findings are consistent with the results from of Prentice *et al*.^5^ and Liu *et al*.^6^, who showed that the serum CMPF levels were higher in T2DM and prediabetes participants. Any differences in age and glycometabolic state could influence the results. Thus, future studies with larger sample sizes should explore the relationship between CMPF and diabetes.

A controversy has emerged regarding the associations between the serum CMPF levels, glucose regulation, and β cell function. A cross-sectional study conducted by Retnakaran *et al*.^7^ analyzed 105 patients with GDM and 290 matched controls. The results showed that the circulating CMPF concentrations were positively associated with AUC_glucose_ and negatively associated with the insulinogenic index/HOMA-IR in women with GDM. Our study demonstrated that the serum CMPF levels were positively correlated with FPG and 2hPG, but negatively correlated with the Stumvoll first-phase insulin secretion index values after adjusting for sex, age, and body mass index. An additional regression analysis showed that the circulating CMPF levels were independently associated with β cell dysfunction. In contrast to the study by Retnakaran *et al*.^7^, our study found that the serum CMPF levels were not significantly associated with insulin sensitively. A recent report by Retnakaran *et al*.^14^ suggested that the serum CMPF levels in pregnant women do not predict postpartum insulin sensitivity, insulin resistance, or β cell function three months after delivery. In addition, Lankinen *et al*.^15^ found that serum CMPF was not associated with any impairment in glucose or insulin metabolism in IGT participants. Age, sex, race, and dietary habits might influence the serum CMPF levels and cause these differences. However, the role of serum CMPF in the etiology of diabetes remains unclear. Studies using rodent models showed that CMPF impaired glucose tolerance and increased the generation of ROS^5^ by altering the activity of AKT^16^ and GSK3β^17^, which are involved in the regulation of insulin transcription^18^. These changes resulted in insulin secretion disorders^5^. Furthermore, blocking the dominant CMPF^19^ transporter OAT3 inhibited CMPF entry into β cells and prevented the destruction of β cell function^5^. These findings suggest that CMPF predicts glucose homeostasis disorders. However, the mechanism of action in humans has not been elucidated.

The pathological basis of T2DM involves β cell dysfunction secondary to adverse environmental factors and genetic susceptibility^20^. FDRs have a higher risk of developing T2DM due genetic and lifestyle factors. Interestingly, our results showed that individuals with FDRs had higher serum CMPF levels than NGT and prediabetes subjects. In humans metabolism of furan fatty acids provided by vegetables, fruit, fish and high-temperature cooking of methyl oleate and linoleate can modestly increase serum CMPF levels^15, 21, 22^. FDRs with normal glucose tolerance may have similar eating habits and lifestyle as T2DM patients, which could contribute to the higher serum CMPF levels. FDRs have higher serum CMPF levels than NGT subjects without a family history of T2DM. These finding suggest that the adverse gluco-regulatory effects of CMPF increase the risk of T2DM in subjects with a potential genetic susceptibility. Several previous studies have shown that non-diabetic FDRs have defects in β cell function^23, 24^. Although the current clinical data suggest that FDRs have normal glucose tolerance and insulin secretion, their β cell function may be damaged. Thus, follow-up studies of FDRs are necessary. Our current findings suggest that eating habits, lifestyle, and genetic susceptibility all contribute to the higher serum CMPF level and increase the risk of T2DM.

The results of our study identified a potential link between serum CMPF and lipid metabolism. We found that circulating CMPF levels were independently and inversely correlated with triglycerides, which is consistent with the results of Zheng *et al*.^13^. Studies using mouse models showed elevated serum CMPF induced by changing the mitochondrial membrane potential decreased glucose oxidation and significantly enhanced fatty acid oxidation. These changes resulted in impaired glucose-stimulated insulin secretion^6^. In our opinion, CMPF might reduce glucose use and increase fatty acid metabolism. The higher use of fatty acids can decrease their serum level. Therefore, we speculate that serum CMPF has an important role in lipid metabolism. However, the pathway has not been elucidated and the mechanism should be explored in further studies.

One major limitation of the current study is its cross-sectional design, which cannot reflect the causal relationship between CMPF and β cell dysfunction. However, earlier studies demonstrated that CMPF directly impairs β cell secretion *in vitro* and *in vivo*. These results support our findings. Additionally, our sample cohort was not large enough and should be expanded in future studies. Furthermore, the postprandial CMPF levels were not measured and only the fasting CMPF values were analyzed. Finally, CMPF was measured using frozen but sufficiently fresh specimens. There were no differences in CMPF levels between frozen and fresh samples.

In conclusion, our results indicate that serum CMPF levels are higher in prediabetes, T2DM, and FDR participants than in NGT controls. The serum CMPF level was independently associated with β cell secretion. β cell function gradually decreased as the CMPF levels increased. Our findings also suggest that serum CMPF might predict glycolipid metabolism disorders and that elevated serum CMPF concentrations could have an important role in the pathogenesis of hyperglycemia and β cell dysfunction.

## Materials and Methods

### Ethics statement

All participants provided written informed consent before participating in this study. The Medical Ethics Committee of the Shanghai Fengxian District Central Hospital approved this study, which complied with the principles of the Declaration of Helsinki as revised in 2000.

### Participants

This study recruited a total of 516 participants, including 99 newly diagnosed cases with T2DM, 219 cases with prediabetes (82 with I − IGT, 66 with I − IFG and 71 with IGT + IFG), and 198 cases with NGT (99 FDRs and 99 non-FDRs). The diagnoses of NGT (3.9 mmol/L ≤ FPG < 5.6 mmol/L), T2DM (FPG ≥ 7.0 mmol/L or 2hPG ≥ 11.1 mmol/L), I − IFG (5.6 mmol/L ≤ FPG < 7.0 mmol/L and 3.9 mmol/L ≤ 2hPG < 7.8 mmol/L), I − IGT (3.9 mmol/L ≤ FPG < 5.6 mmol/L and 7.8 mmol/L ≤ 2hPG ≤ 11.0 mmol/L) and IGT + IFG (5.6 mmol/L ≤ FPG < 7.0 mmol/L and 7.8 mmol/L ≤ 2hPG ≤ 11.0 mmol/L) were based on the ADA 2003 criteria. The following exclusion criteria were used: histories of diabetes, acute or chronic inflammatory disease, heart, liver or renal failure cancer, or active use of oral hypotensive, hypolipidemic, anti-diabetic, or other medications known to affect glycolipid metabolism. The sample size was evaluated based on our pilot study and the report from Liu *et al*.^6^. The minimal required sample size in each group was assessed according to the difference CMPF levels between the T2DM and NGT groups (mean difference = 59.4, SD_1_ = 67.08, SD_2_ = 160.35). The criteria of a type I error was set at α = 0.05 and type II error was set at β = 0.10 using the MedCalc software (n = 91 per group).

### Clinical measurements

The height (without shoes) and weight (in light clothing) were measured, and the body mass index was calculated as weight (kilograms) divided by the square of height (square meters). All blood samples were collected from the antecubital vein following 8 h of overnight fasting. An oral glucose tolerance test (OGTT) was conducted on all participants, and blood samples were obtained after 2 h. After clotting, the serum samples were separated from the blood specimens via centrifugation for 15 min at 1,000 *g* and stored in aliquots at −80 °C until CMPF analysis. The blood total cholesterol, triglycerides, low-density lipoprotein cholesterol (LDL-c), high-density lipoprotein cholesterol (HDL-c), free fatty acid, fasting plasma glucose (FPG), and 2-h postprandial glucose (2hPG) were measured using an automatic biochemical analyzer (Beckman DXC800, USA). The fasting insulin (FINS) and 2-h postprandial insulin (2hINS) levels were determined using a Roche-Elecsys-1010 immunoassay analyzer and electrochemiluminescence immunoassay kit (Roche Diagnostics, Germany). The hemoglobin A1C (HbA1C) was measured using high-pressure liquid chromatography (TOSOH HLC-723 G7, Japan).

The homeostasis model assessment of insulin resistance (HOMA-IR) was calculated as FPG (mmol/L) × FINS (mU/L)/22.5. The homeostasis model assessment-β (HOMA-β) was calculated as 20 × FINS (mU/L)/(FPG [mmol/L] − 3.5). The Stumvoll first- and second-phase insulin secretion indices were calculated using the following equations: 2,032 + 4.681 × FINS (pmol/L) − 135.0 × 2hPG (mmol/L) + 0.995 × 2hINS (pmol/L) + 27.99 × body mass index (kg/m^2^) − 269.1 × FPG (mmol/L) and 277 + 0.800 × FINS (pmol/L) − 42.79 × 2hPG (mmol/L) + 0.321 × 2hINS (pmol/L) + 5.338 × body mass index (kg/m^2^), respectively.

### Serum CMPF measurement

The serum CMPF concentrations were determined using an enzyme-linked immunosorbent assay (ELISA) kit #BG-HUM10440 (catalog number 072204KB; Novateinbio, Inc., USA). All serum samples were analyzed without dilution. A standard curve was constructed by plotting the absorbance at 450 nm versus the CMPF concentrations of the calibrators. The sample concentrations were determined using the standard curve. The lower and upper limits of detection of the human ELISA were 0 and 1,000 ng/ml, respectively.

### Statistics

All analyses were performed using SPSS software version 19.0. The data are presented as the means ± SD or medians (interquartile ranges). The Kolmongorov-Smirnov test was used to explore the normality of the whole data set prior to analysis. One-way ANOVAs and Kruskal-Wallis H(K) tests were used to compare the differences among the four groups for normally and abnormally distributed variables, respectively. Student-Newman-Keuls and Mann-Whitney *U* tests were performed to compare the differences between any two of the six groups for normally and abnormally distributed variables, respectively. CMPF, triglycerides, FPG, 2hPG, HbA1c, FINS, 2hINS, HOMA-β, HOMA-IR, and Stumvoll first- and second-phase insulin secretion index values were logarithmically transformed due to their abnormal distribution. After this transformation, Pearson’s bivariate correlation analyses were used to explore the correlations between CMPF and the clinical parameters. Various parameters, including body mass index, triglycerides, and the Stumvoll first- and second-phase insulin secretion index values, were included in a multiple linear regression analysis to determine the contributions of these variables to CMPF. The alpha level for all tests was 0.05. A two-sided P-value of <0.05 was considered as significant.

### Data availability

Due to the sensitive nature of the data and consent agreements signed by participants, the data cannot be made publicly available. The data are available upon request. Please include a proposal for use of the data. The proposal will be submitted to the authors’ ethics committee for approval. Requests for these data may be sent to xuemeiyu12@163.com. The authors confirm that all of the interested parties will be able to obtain these data via the contact provided after the appropriate documentation is completed.
